# Estrogen Modulates Expression of Tight Junction Proteins in Rat Vagina

**DOI:** 10.1155/2016/4394702

**Published:** 2016-04-05

**Authors:** Kyung-Jin Oh, Hyun-Suk Lee, Kyuyoun Ahn, Kwangsung Park

**Affiliations:** ^1^Department of Urology, Chonnam National University Medical School, Sexual Medicine Research Center, Chonnam National University, Gwangju 61469, Republic of Korea; ^2^Department of Anatomy, Chonnam National University Medical School, Sexual Medicine Research Center, Chonnam National University, Gwangju 61469, Republic of Korea

## Abstract

*Background*. The objectives of this study were to investigate the localization of tight junctions and the modulation of zonula occludens- (ZO-) 1, occludin and claudin-1 expression by estrogen in castrated female rat vagina. Female Sprague-Dawley rats (230–240 g, *n* = 45) were divided into three groups and subjected to a sham operation (control group, *n* = 15), bilateral ovariectomy (Ovx group, *n* = 15), or bilateral ovariectomy followed by daily subcutaneous injection of 17*β*-estradiol (50 *μ*g/kg/day, Ovx + Est group, *n* = 15). The cellular localization and expression of ZO-1, occludin, and claudin-1 were determined in each group by immunohistochemistry and western blot.* Results*. Expression of ZO-1 was diffuse in all groups, with the highest intensity in the superficial epithelium in the control group. Occludin was localized in the intermediate and basal epithelium. Claudin-1 was most intense in the superficial layer of the vaginal epithelium in the control group. Expression of ZO-1, occludin, and claudin-1 was significantly decreased after ovariectomy and was restored to the level of the control after estrogen replacement.* Conclusions*. Tight junctions are distinctly localized in rat vagina, and estrogen modulates the expression of tight junctions. Further researches are needed to clarify the functional role of tight junctions in vaginal lubrication.

## 1. Introduction

One of the consequences of sexual arousal in the female is physiological changes in the vagina, including genital engorgement, genital swelling, and vaginal lubrication [1]. The exact mechanism of vaginal lubrication is not yet clearly understood. The vaginal wall consists of the mucosal epithelial stratum, a lamina propria containing veins, the muscularis stratum, and the external adventitial layer [2]. The mucosa of the vagina consists of stratified squamous epithelium devoid of glands [3]. Although the vagina does not have glands, it secretes vaginal fluid during sexual arousal [4]. The main contributor to this lubrication is capillary plasma transudate. An active interaction takes place between the microvasculature and the epithelial cells of the vaginal wall [5]. Thus, the mechanism of vaginal lubrication has been suggested to be a consecutive event in which plasma leaks out of the capillaries into the vaginal epithelium [6].

The route of transport in the vaginal epithelia could be transcellular or paracellular [7]. Several studies reported on transcellular transport via aquaporin (AQP) water channels in the vagina [6, 8]. However, paracellular transport mechanisms are not well elucidated. Transport in the paracellular pathway is controlled mainly by the tight junction [9]. Paracellular permeability is a key factor in vaginal lubrication. Epithelial intracellular junctions have specific combinations of specialized molecules. Tight junctions seal the space between adjacent epithelial cells and prevent free movement of molecules through the paracellular space [10]. Tight junctions consist of a network of transmembrane proteins, such as occludin, claudins, and junctional adhesion molecules [11]. These proteins are linked to the cytoskeleton by zonula occludens (ZO) proteins, which serve as regulatory proteins to the tight junction [12].

Estrogen has an important role in regulating epithelial permeability and in controlling fluid transudation into the cervical and vaginal lumens. Estrogen lessens capillary resistance and increases blood flow and tends to increase the subluminal to luminal hydrostatic gradient. Also, estrogen can modulate epithelial permeability through regulation of the resistance of the epithelial tight junctions [13].

We hypothesized that the expression of tight junctions would be affected by estrogen deprivation and restored by estrogen supplementation. The main objective of our study was therefore to investigate the expression of tight junctions and the modulation of expression by estrogen in rat vagina.

## 2. Materials and Methods

### 2.1. Experimental Animals

Female Sprague-Dawley rats (230–240 g, *n* = 45) were divided into three groups and subjected to a sham operation (control group, *n* = 15), bilateral ovariectomy (Ovx group, *n* = 15), or bilateral ovariectomy followed by subcutaneous injections of 17*β*-estradiol (Ovx + Est group, *n* = 15). The Ovx group underwent bilateral ovariectomy and received injections of oil vehicle only. The Ovx + Est group underwent bilateral ovariectomy followed by daily subcutaneous injections of 17*β*-estradiol (50 *μ*g/kg/day; Sigma Chemical Co., St. Louis, MO, USA). Replacement of 17*β*-estradiol was begun at 7 days after the ovariectomy. Four weeks after the operation, animals were premedicated with xylazine (2.2 mg/kg, intramuscular) and anesthetized with a zolazepam/tiletamine cocktail (4.4 mg/kg, intramuscular). The lower one-third of the vaginal tissue was cleanly dissected from both lateral walls in all three groups. All experimental procedures were performed according to the guidelines of the animal ethics committee of the Chonnam National University Medical School and were approved by the committee.

### 2.2. Immunohistochemical Staining

The vaginal tissue was placed in 4% paraformaldehyde fixative for 16 hours and then processed for washing and dehydration. The tissues were embedded in paraffin. Sections of 5 *μ*m thickness were prepared for staining. Immunohistochemistry was performed by using an immunoperoxidase procedure (Vector ABC Kit; Vector Laboratories, Burlingame, CA, USA). The tissue sections were deparaffinized in xylene, dehydrated in a graded series of ethanol, rinsed twice in phosphate-buffered saline (PBS), and then treated with 3% H_2_O_2_ in 60% methanol for 30 minutes to quench endogenous peroxidase activity. After washing twice (5 minutes) in PBS, the sections were incubated for 12 to 14 hours with antibodies to ZO-1 (1 : 200, Invitrogen), occludin (1 : 200, Zymed), and claudin-1 (1 : 200, Invitrogen) in PBS with 0.3% bovine serum albumin. For a negative control, the sections were incubated in PBS containing 5% normal goat serum only. The sections were then rinsed 3 times in PBS and incubated sequentially for 30 minutes each with the biotinylated secondary antibody and the ABC reagent. The sections were incubated for 5 minutes with a peroxidase substrate solution (diaminobenzidine). Finally, the tissue sections were examined and photographed under a light microscope.

### 2.3. Western Blot

Vaginal tissues were homogenized in phosphate buffer (10-mM phosphate [pH 7.4] and 150 mM NaCl) containing protease inhibitors (1 mM phenylmethylsulfonyl fluoride, 25 *μ*g/mL aprotinin, and 25 *μ*g/mL leupeptin). The homogenates were centrifuged at 12,000 rpm for 20 minutes. The total protein concentration in the supernatant was measured by the Lowry micromethod. Equal amounts of protein (50 *μ*g) were electrophoresed on sodium dodecyl sulfate- (SDS-) 10% polyacrylamide gels and transferred to a polyvinylidene difluoride membrane (Amersham Pharmacia Biotech, Little Chalfont, United Kingdom). The blots were then washed with Tris-buffered saline Tween-20 (10 mM Tris-HCl, pH 7.6, 150 mM NaCl, 0.05% Tween-20). The membrane was blocked with 5% skimmed milk for 1 hour and incubated with the appropriate primary antibody at the dilution recommended by the supplier. ZO-1 (1 : 1000, Invitrogen), occludin (1 : 1000, Zymed), and claudin-1 (1 : 1000, Invitrogen) were used. The membrane was then washed. The primary antibodies were detected with goat anti-rabbit-IgG conjugated with horseradish peroxidase. The bands were visualized by using enhanced chemiluminescence (Amersham Pharmacia Biotech). *β*-Actin was used as a reference. Densitometry analysis was performed with a studio star scanner (Agfa-Gevaert, Mortsel, Belgium).

### 2.4. Data Analysis

Data were expressed as mean ± standard error of the mean. The significance of differences among the groups was determined by using Student's *t*-test and ANOVA, with differences considered significant at *P* < 0.05.

## 3. Results

### 3.1. Immunohistochemistry of Zonula Occludens- (ZO-) 1, Occludin, and Claudin-1

**Figure 1:**
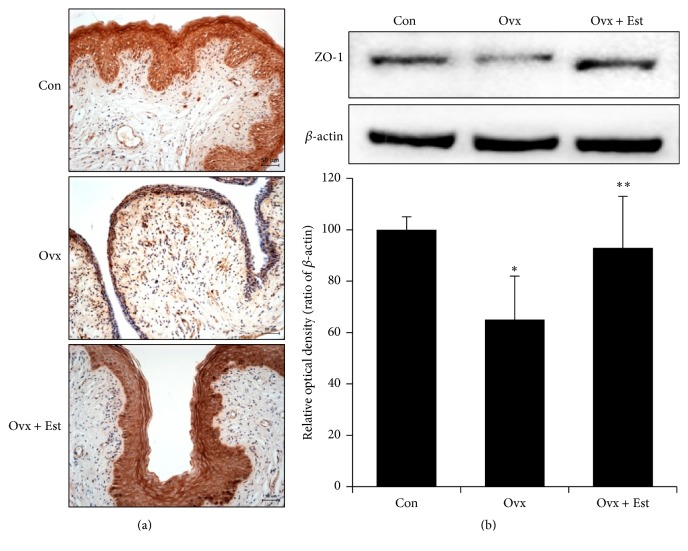
(a) Immunohistochemistry of zonula occludens- (ZO-) 1 in vaginal tissue from animals of the control (Con), ovariectomy (Ovx), and ovariectomy plus 17*β*-estradiol treatment (Ovx + Est) groups. ZO-1 was mainly expressed in the superficial epithelium. (b) Immunoblotting of ZO-1 in the rat vagina. The lower panels denote the means ± standard error of 6 experiments for each condition determined by densitometry relative to beta-actin. ^*∗*^ *P* < 0.05 versus control. ^*∗∗*^ *P* < 0.05 versus Ovx.

**Figure 2:**
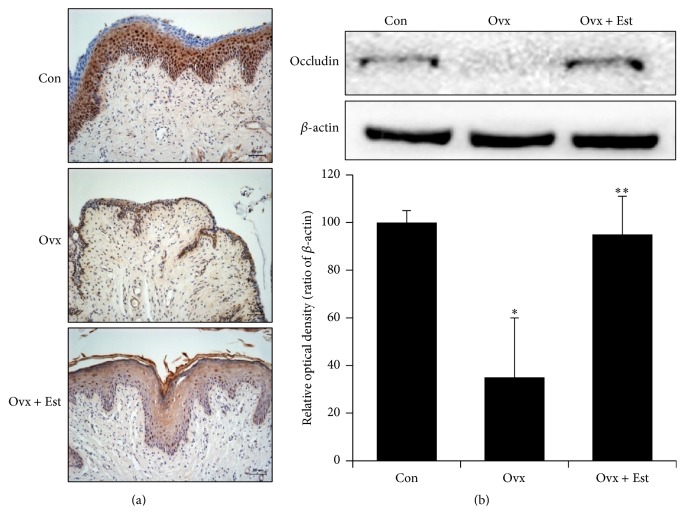
(a) Immunohistochemistry of occludin in vaginal tissue from animals of the control (Con), ovariectomy (Ovx), and ovariectomy plus 17*β*-estradiol treatment (Ovx + Est) groups. Occludin was expressed in the intermediate and basal epithelium. (b) Immunoblotting of occludin in the rat vagina. The lower panels denote the means ± standard error of 6 experiments for each condition determined by densitometry relative to beta-actin. ^*∗*^ *P* < 0.05 versus control. ^*∗∗*^ *P* < 0.05 versus Ovx.

**Figure 3:**
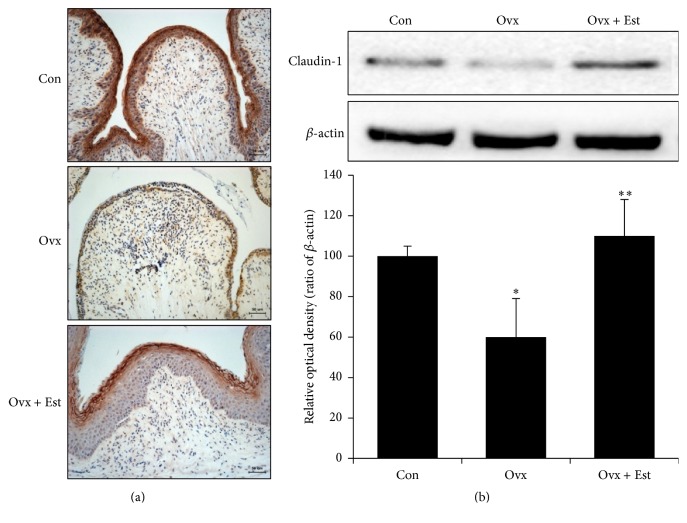
(a) Immunohistochemistry of claudin-1 in vaginal tissue from animals of the control (Con), ovariectomy (Ovx), and ovariectomy plus 17*β*-estradiol treatment (Ovx + Est) groups. Claudin-1 was most intense in the superficial layer of the vaginal epithelium. (b) Immunoblotting of claudin-1 in the rat vagina. The lower panels denote the means ± standard error of 6 experiments for each condition determined by densitometry relative to beta-actin. ^*∗*^ *P* < 0.05 versus control. ^*∗∗*^ *P* < 0.05 versus Ovx.

Vaginal tissue from the control group showed a multiple-layered epithelium. After ovariectomy, the vaginal tissue showed atrophic changes, with a thinner epithelium and mucosal distortion. After treatment with 17*β*-estradiol, the thickness of the vaginal epithelium was restored and the epithelium looked similar to the control tissue (Figures 1(a), 2(a), and 3(a)).

Expression of ZO-1 was diffusely localized, and the intensity was highest in the superficial layer of the epithelium in the control group (Figure 1(a)). ZO-1 protein expression was significantly lower after ovariectomy and was restored to the level of the control after 17*β*-estradiol treatment (Figure 1(b)). Expression of occludin was localized in the intermediate and basal epithelium. Occludin was not detected in the superficial layer of the vagina in the control group (Figure 2(a)). Expression of occludin protein significantly decreased after ovariectomy but was restored to the level of the control after 17*β*-estradiol treatment (Figure 2(b)). Expression of claudin-1 was most intense in the superficial layer of the vaginal epithelium in the control (Figure 3(a)). After estradiol injection, claudin-1 was less intense throughout the epithelium, with a region of strong expression in the outer superficial epithelium (Figure 3(a)).

### 3.2. Immunoblotting of Zonula Occludens- (ZO-) 1, Occludin, and Claudin-1

Western blot analysis showed that claudin-1 protein expression was significantly lower in the ovariectomy group than in the control group and was restored to the level of the control group after 17*β*-estradiol treatment (Figure 3(b)). Thus, the expression of all tight junctions showed a similar pattern with a decrease after ovariectomy and restoration of expression after 17*β*-estradiol injection.

## 4. Discussion

Vaginal lubrication is a key phenomenon in female genital sexual arousal. The major route of transport in the vaginal epithelia could be transcellular or paracellular [7]. The transcellular transport via AQP in the vagina has been well investigated [6, 8]. However, paracellular transport mechanisms are not well elucidated.

Paracellular transport of fluid is controlled by the resistance of the epithelial tight junctions and epithelial lateral intercellular space [14, 15]. The gate of the tight junction has a relatively high resistance that is determined by occlusion of the intercellular space through the interactions of extracellular loops of transcellular tight junction proteins [13, 16, 17]. The transmembrane proteins restrict the paracellular diffusion of molecules across the epithelial sheet [11]. Tight junction complexes are composed of 3 classes. The main framework consists of transmembrane-spanning proteins such as occludin and the various claudin family members. Cytoskeletal proteins are involved in regulating the permeability of tight junctions. Cytosolic plaque proteins such as ZO-1 connect the transmembrane proteins to the actin cytoskeleton. In the present study, we investigated the expression of ZO-1, occludin, and claudin-1 and the modulation of expression by estrogen in rat vagina.

In this study, we found that tight junctions were distinctly localized in rat vagina. The expression of ZO-1 was diffusely localized, and the intensity was highest in the superficial layer of the epithelium. And the expression of claudin-1 was most intense in the superficial layer of the vaginal epithelium. However, the expression of occludin was localized in the intermediate and basal epithelium of the vagina.

The tight junction protein pattern in the different cell layers can be highly variable. In bovine gingiva, claudin-1-positive tight junction structures are seen in all suprabasal layers, together with ZO-1, whereas occludin is found only in the uppermost strata [11]. Various tight junction protein reactions in different numbers of regional and cell-type-specific layers have been reported in vagina [18]. In stratified squamous epithelia, tight junction proteins are not restricted to the ZO-related structures of the uppermost living cell layer of the epidermis [19]. Schlüter et al. showed that tight junction membrane proteins such as occludin, claudin, and ZO-1 are expressed in more basal layers where they form special cell-cell connecting structures [19]. In human vagina, no tight junction molecules are detected in the apical layers of the vaginal epithelia [12]. Instead, tight junction molecules are localized to the lower two-thirds of the epithelium in a spider web-like pattern [12].

Estrogen has an important role in regulating epithelial permeability and controlling fluid transudation in the cervix and vagina. Transepithelial transport is basically understood as a leaky phenomenon in the cervical and vaginal epithelia [14, 15]. Estrogen controls fluid transudation in several proposed ways. Estrogen decreases capillary resistance and then increases blood flow, which increases the subluminal to luminal hydrostatic gradient. The main estrogenic control of transepithelial transport is by modulation of epithelial permeability through regulation of the resistance of tight junctions and the lateral intercellular space [13]. It is well known that estrogen decreases the resistance of tight junctions by matrix metalloproteinase-7-dependent modulation of occludin [9, 20]. The decrease in resistance of the tight junction occurs through a loss of gating of the intercellular space following degradation of occludin extracellular loops. In an experiment using normal human epithelial vaginal-ectocervical cells from premenopausal and postmenopausal women, overall epithelial permeability was shown to decrease after menopause and to lead to decreased plasma transudation and decreased lubrication of the lumen [13]. In the present study, we investigated the expression of tight junction proteins in ovariectomized rat vagina and the changes in these proteins in response to estrogen. The expressions of ZO-1, occludin, and claudin-1 were downregulated after ovariectomy and upregulated to the control level after 17*β*-estradiol treatment, respectively. These findings suggest that tight junctions are influenced by changes in estrogen hormones. This could explain the development of vaginal dryness in menopause and may suggest a therapeutic strategy for managing menopausal symptoms. A limitation of the present study is that we could not clarify whether increased expression of tight junction proteins is related to changes in tight junction structures. Further researches are needed to clarify the functional role of tight junctions in vaginal lubrication.

## 5. Conclusions

Our results have demonstrated that tight junction molecules are well expressed in rat vagina. ZO-1, occludin, and claudin-1 are expressed in distinct locations. Estrogen deprivation resulted in a significant downregulation of the expression of tight junctions. Furthermore, estrogen replacement in ovariectomized rats resulted in upregulation of tight junction proteins to the control level. Our results thus show that estrogen may play an important role in modification of tight junction expression. Further researches are needed to clarify the functional role of tight junctions in vaginal lubrication.

## References

[1] Levin R. J. (1992). The mechanisms of human female sexual arousal. *Annual Review of Sex Research*.

[2] Jannini E. A., d’Amati G., Lenzi A., Goldstein I., Meston C. M., Davis S. R., Traish A. M. (2006). Histology and immunohistochemical studies of female genital tissue. *Women's Sexual Function and Dysfunction*.

[3] Levin R. J. (1980). The physiology of sexual function in women. *Clinics in Obstetrics and Gynaecology*.

[4] Munarriz R., Kim N. N., Goldstein I., Traish A. M. (2002). Biology of female sexual function. *Urologic Clinics of North America*.

[5] Shabsigh A., Buttyan R., Burchardt T. (1999). The microvascular architecture of the rat vagina revealed by image analysis of vascular corrosion casts. *International Journal of Impotence Research*.

[6] Kim S.-O., Lee H.-S., Ahn K., Park K. (2009). Effect of estrogen deprivation on the expression of aquaporins and nitric oxide synthases in rat vagina. *Journal of Sexual Medicine*.

[7] Salama N. N., Eddington N. D., Fasano A. (2006). Tight junction modulation and its relationship to drug delivery. *Advanced Drug Delivery Reviews*.

[8] Park K., Han H. J., Kim S. W. (2008). Expression of aquaporin water channels in rat vagina: potential role in vaginal lubrication. *Journal of Sexual Medicine*.

[9] Gorodeski G. I. (2007). Estrogen decrease in tight junctional resistance involves matrix-metalloproteinase-7-mediated remodeling of occludin. *Endocrinology*.

[10] Kondoh M., Takahashi A., Yagi K. (2012). Spiral progression in the development of absorption enhancers based on the biology of tight junctions. *Advanced Drug Delivery Reviews*.

[11] Langbein L., Grund C., Kuhn C. (2002). Tight junctions and compositionally related junctional structures in mammalian stratified epithelia and cell cultures derives therefrom. *European Journal of Cell Biology*.

[12] Blaskewicz C. D., Pudney J., Anderson D. J. (2011). Structure and function of intercellular junctions in human cervical and vaginal mucosal epithelia. *Biology of Reproduction*.

[13] Gorodeski G. I. (2007). Estrogen modulation of epithelial permeability in cervical-vaginal cells of premenopausal and postmenopausal women. *Menopause*.

[14] Ussing H. H., Zerahn K. (1951). Active transport of sodium as the source of electric current in the short-circuited isolated frog skin. *Acta Physiologica Scandinavica*.

[15] Spring K. R., Hope A. (1978). Size and shape of the lateral intercellular spaces in a living epithelium. *Science*.

[16] Furuse M., Fujita K., Hiiragi T., Fujimoto K., Tsukita S. (1998). Claudin-1 and -2: novel integral membrane proteins localizing at tight junctions with no sequence similarity to occludin. *Journal of Cell Biology*.

[17] Furuse M., Hirase T., Itoh M. (1993). Occludin: a novel integral membrane protein localizing at tight junctions. *Journal of Cell Biology*.

[18] Franke W. W., Pape U.-F. (2012). Diverse types of junctions containing tight junction proteins in stratified mammalian epithelia. *Annals of the New York Academy of Sciences*.

[19] Schlüter H., Moll I., Wolburg H., Franke W. W. (2007). The different structures containing tight junction proteins in epidermal and other stratified epithelial cells, including squamous cell metaplasia. *European Journal of Cell Biology*.

[20] Zeng R., Li X., Gorodeski G. I. (2004). Estrogen abrogates transcervical tight junctional resistance by acceleration of occludin modulation. *Journal of Clinical Endocrinology and Metabolism*.

